# Hedgehog-glioma-associated oncogene homolog-1 signaling in colon cancer cells and its role in the celecoxib-mediated anti-cancer effect

**DOI:** 10.3892/ol.2014.2439

**Published:** 2014-08-12

**Authors:** HONGTAO WANG, FEI KE, JIE ZHENG

**Affiliations:** Department of Pathology, School of Medicine, Southeast University, Nanjing, Jiangsu 210009, P.R. China

**Keywords:** colon cancer cells, hedgehog signaling, celecoxib, cyclopamine, glioma-associated oncogene homolog-1

## Abstract

Hedgehog (Hh) signaling is activated in numerous malignant tumors, but its role in human colorectal cancer remains uncertain. Celecoxib, a selective cyclooxygenase-2 inhibitor, has been shown to exhibit chemoprevention in colorectal cancer, however, the effects of celecoxib on Hh signaling remain unknown. The current study presents an evaluation of Hh signaling in colon cancer cell lines and the effects of celecoxib *in vitro*. Active Hh signaling was observed in LoVo and HT-29 cells, with particularly high levels in the LoVo cells. However, Hh signaling activity was absent in HCT-116 cells. Quantitative polymerase chain reaction indicated that the expression of Hh receptor patched homolog 1 (*PTCH1*) was absent in the LoVo cells, but that they exhibited high levels of glioma-associated oncogene homolog-1 (*GLI1*) expression, while high expression levels of *PTCH1* and low expression levels of smoothened (*SMO*) and *GLI1* were observed in the HCT-116 cells. The HCT-116 cells were extremely sensitive to celecoxib, whereas the LoVo cells were resistant to the anticancer effect of the drug. Celecoxib downregulated the expression of *GLI1* in the HCT-116 and HT-29 cells, but did not change the expression of *GLI1* in the LoVo cells. The results presented in this study indicated that the anticancer effect of celecoxib is selective in colon cancer cells; celecoxib may target cancer cells via the SMO-independent modulation of GLI1 activity, and Hh signaling may be significant in maintaining the malignant state of LoVo cells. These findings may aid in improving our understanding of the carcinogenesis of colon cancer and the development of novel approaches for the targeted therapy of this disease.

## Introduction

Colorectal cancer is one of the most common malignancies worldwide, with >1,000,000 cases reported annually (1). Several developmental signaling pathways that are involved in the carcinogenesis of colorectal cancer, including the Wnt/β-catenin (2), TGF-β/Smad (3) Notch (4) and receptor tyrosine kinase (5) pathways, have been widely investigated. However, the role of hedgehog (Hh)-glioma-associated oncogene homolog (GLI) signaling in colorectal cancer remains controversial (6,7), and certain studies have indicated that Hh signaling is inactive in colorectal cancer (8–10).

Canonical Hh signaling predominantly consists of Hh, the Hh receptor, patched homolog 1 (PTCH1), the intermediary signaling molecule, smoothened (SMO), and the transcription factor, GLI. The mammalian GLI family has three isoforms, GLI1, GLI2 and GLI3. GLI1 is an activator of primary transcription, whereas GLI3 is a repressor of transcription and GLI2 has the functions of a transcriptional activator and repressor (11). In the absence of Hh, PTCH1 interacts with SMO and inhibits its activity, while GLI is degraded to the repressor form, which results in the transcriptional inhibition of Hh target genes. When an Hh ligand binds to the PTCH1 receptor, it relieves the PTCH1-mediated inhibition of SMO. Subsequently, the active SMO affects the expression of GLI proteins, which may enter the nucleus and regulate the expression of the Hh target genes (including *PTCH1*, *GLI*, *Wnt*, *c-MYC* and *CCND1*) in responding cells (12,13). Aberrant Hh signaling was initially identified in nevoid basal cell carcinoma syndrome, also termed Gorlin syndrome (14). Soon afterwards, aberrant Hh signaling activity was also observed in several types of human cancer, including medulloblastoma, glioblastoma, rhabdomyosarcoma, pancreatic and prostate cancer, and hematological malignancies (15). Cyclopamine, a specific inhibitor of SMO (16), is currently under investigation in anticancer studies.

Cyclooxygenase-2 (COX-2) has been reported to be highly expressed in a number of human cancers and cancer cell lines, including pancreatic and colon cancer (17). Celecoxib, a selective COX-2 inhibitor, may inhibit the proliferation of cancer cells and promote apoptosis (18). In addition, celecoxib has been approved by the Food and Drug Administration in the USA for the chemoprevention of colorectal cancer. At present, it is unclear whether celecoxib and its anticancer effects are associated with Hh signaling. The present study aimed to investigate Hh signaling in colon cancer cell lines and the effect of celecoxib on Hh signaling in colon cancer cells.

## Materials and methods

### Cell culture and reagents

The human colon cancer HT-29 and LoVo cell lines, and the pancreatic cancer PANC-1 cell line were obtained from the Cell Bank of Type Culture Collection of Chinese Academy of Sciences (Shanghai Institute of Cell Biology, Chinese Academy of Sciences, Shanghai, China), while the human colon cancer HCT-116 cell line was donated by the experimental center of the Shanghai Traditional Chinese Medicine Hospital (Shanghai, China). The HT-29 cells were cultured in RPMI 1640 medium with 10% newborn calf serum, the LoVo and HCT-116 cells were cultured in RPMI 1640 medium with 10% fetal bovine serum (FBS) and the PANC-1 cells were cultured in Dulbecco’s modified Eagle’s medium with 10% FBS. All culture media also contained 100 U/ml penicillin and 100 μg/ml streptomycin. The PANC-1 cells were used as control cells, as Hh signaling is active in these cells (9). Cyclopamine was purchased from Sigma-Aldrich (St. Louis, MO, USA) and celecoxib was purchased from Pfizer (New York, NY, USA). For the experiments conducted in the present study, these agents were dissolved in dimethyl sulfoxide (DMSO) and were then added to the cells in the corresponding medium with a final DMSO concentration of ≤0.1%.

### Cell viability

A total of 5×10^3^ cells per well were seeded in 96-well plates, and each group consisted of five parallel wells. Following 24 h of incubation, fresh medium was added to the cells, with or without cyclopamine or celecoxib. Following the required period of culture, cell viability was determined by MTT assay according to the following formula: Cell viability (%) = [optical density (OD)_average dosing group_ / OD_control group mean_] × 100.

### Measurement of GLI1 levels

A total of 2×10^5^ cells per well were seeded in six-well plates, and each group consisted of two parallel wells. At 24 h after the seeding, the cells were treated with cyclopamine or celecoxib. The cells were then harvested following the required period of treatment and nuclear proteins were subsequently extracted from the treated cells using a nuclear protein/plasma protein extraction kit (Aidlab Biotechnologies Co. Ltd., Beijing, China). The protein concentrations were quantified using the bicinchoninic acid protein quantitative kits (Beyotime Institute of Biotechnology, Jiangsu, China) according to the manufacturer’s instructions. The levels of GLI1 were measured by ELISA (CUSABIO Biotech Co., Ltd., Wuhan, China).

### Expression of PTCH1, SMO and GLI1 genes by quantitative polymerase chain reaction (qPCR)

In total, 2×10^5^ cells per well were seeded in six-well plates, and treated with cyclopamine or celecoxib for 36 h. Total RNA was isolated using TRIzol (Invitrogen Life Technologies, Carlsbad, CA, USA) according to the manufacturer’s instructions. Next, the expression of the mRNA was examined by qPCR using the StepOnePlus™ Real-Time PCR System (Applied Biosystems, Foster City, CA, USA) and Fast SYBR Green Master Mix 2X reagent (Applied Biosystems, Foster City, CA, USA) in a 20 μl reaction volume according to the manufacturer’s instructions. The thermal cycling conditions were as follows: 20 sec at 95°C, followed by the amplification reaction consisting of 40 cycles of denaturation for 3 sec at 95°C and annealing for 30 sec at 60°C. For sample analysis, the threshold was set based on the exponential phase of the products, and the cycle threshold (Ct) value for the sample was determined. The results were analyzed using the comparative Ct method for relative gene expression quantification against the housekeeping gene, *GAPDH*. The primers were designed using the Oligo Primer Analysis 4.0 software and the sequences were BLASTed (http://www.ncbi.nlm.nih.gov/BLAST/). The primer sequences were as follows: Sense, 5′-GGTGGCACAGTCAAGAACA-3′ and antisense, 5′-TCGTGGTGGTGAAGGAAA-3′ for *PTCH1*; sense, 5′-CCCTTGGTTCGGACAGACA-3′ and antisense, 5′-AAAGAAGCACGCATTGACG-3′for *SMO*; sense, 5′-TTCCTACCAGAGTCCCAAGT-3′ and antisense, 5′-CCCTATGTGAAGCCCTATTT-3′ for *GLI1*; sense, 5′-AACGGATTTGGTCGTATTG-3′ and antisense, 5′-GGA AGATGGTGATGGGATT-3′ for *GAPDH*.

### Statistical analysis

All experiments were performed in duplicate or more. Data are presented as the mean ± standard deviation and the difference between two groups was assessed using Student’s two-tailed t-test. P<0.05 and P<0.01 were considered to indicate statistically significant differences.

## Results

### Effect of cyclopamine or celecoxib on the proliferation of colon cancer cells

**Figure 1:**
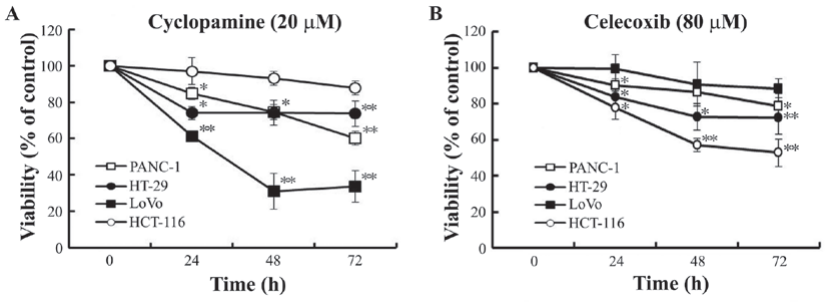
Inhibitory effects of (A) cyclopamine and (B) celecoxib on cancer cell viability. PANC-1 and colon cancer cells were treated with the drug or vehicle for the indicated time periods, and the cells were harvested for MTT assay. The assay was repeated three times and similar results were obtained. The data are presented as the mean of pentaplicates ± standard deviation. ^*^P<0.05 vs. control group, ^**^P<0.01 vs. control group.

As shown in Fig. 1A, the control PANC-1 cells were sensitive to cyclopamine; the rate of inhibition was 15.2% (P<0.05) after 24 h and increased to 39.8% (P<0.01) at 72 h. However, the LoVo cells were more sensitive to the growth inhibition of cyclopamine compared with the PANC-1 cells; the rate of inhibition was ~38.7% (P<0.01) after 24 h and increased to 66.1% (P<0.01) after 72 h. The HT-29 cells were not as sensitive to cyclopamine when compared with the LoVo cells; following 24 and 72 h of incubation, the rate of inhibition was 25.9 (P<0.05) and 26.2% (P<0.01), respectively. The response of the HCT-116 cells to cyclopamine was weak, with a maximum inhibition rate of 12.2% (P>0.05) at 72 h.

The MTT assay results for celecoxib are shown in Fig. 1B. Significant inhibition was observed in the HCT-116 cells; following 24 and 48 h of treatment, the rate of inhibition was 22.2 (P<0.05) and 47.3% (P<0.01), respectively. However, the inhibition of celecoxib was weaker in the HT-29 and PANC-1 cells than in the HCT-116 cells; the rate of inhibition in the HT-29 and PANC-1 cells was 27.6 (P<0.01) and 21.2% (P<0.05), respectively, after 72 h of treatment. The LoVo cells were resistant to the growth inhibition of celecoxib and the maximum inhibition rate was only 11.6% (P>0.05) following 72 h of treatment.

### Effect of cyclopamine or celecoxib on GLI1 level in colon cancer cells

**Figure 2:**
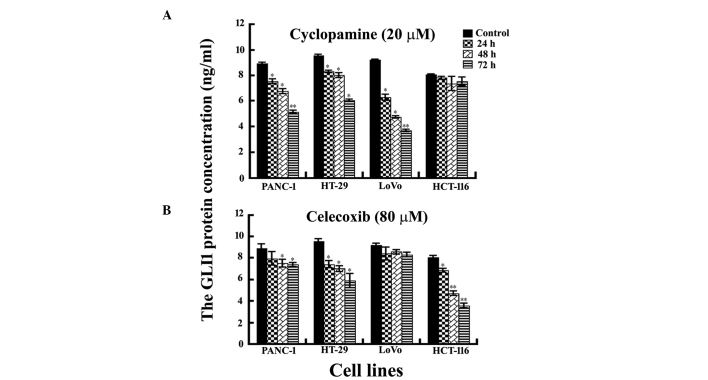
Effects of cyclopamine or celecoxib on the expression of GLI1 in PANC-1 and colon cancer cells. Cells were treated with (A) cyclopamine or (B) celecoxib for the indicated time periods. GLI1 in the cell nuclei was measured by ELISA. The assay was performed in duplicate and similar results were obtained. The data are presented as the mean ± standard error of the mean. ^*^P<0.05 and ^**^P<0.01 vs. control group. GLI1, glioma-associated oncogene homolog-1.

When the PANC-1 cells were treated with cyclopamine, the GLI1 level significantly decreased. Following 24 and 72 h of treatment, the level of GLI1 had decreased by 15.5 (P<0.05) and 42.3% (P<0.01), respectively (Fig. 2A). When the colon cancer cells were treated with cyclopamine, changes were observed in the GLI1 levels between the cell lines (Fig. 2A). The effect of cyclopamine on GLI1 was pronounced in the LoVo cells, consistent with the MTT results (Fig. 1A). Following 24 and 72 h of treatment, the level of GLI1 had declined by 31.6 (P<0.05) and 59.9% (P<0.01), respectively. The HT-29 cells were the second most sensitive to cyclopamine from the three colon cancer cell lines; the GLI1 levels were decreased by 36.4% (P<0.05) after 72 h. However, the HCT-116 cells were not sensitive to cyclopamine; the GLI1 levels were only decreased by 6.4% (P>0.05), consistent with the MTT results (Fig. 1A).

When the colon cancer cells were treated with celecoxib, changes in the GLI1 levels in the three cell lines were evident (Fig. 2B). The level of GLI1 was significantly decreased in the HCT-116 cells, consistent with the MTT results (Fig. 1B); following 24 and 72 h of treatment, the GLI1 levels had decreased by 14.8 (P<0.05) and 55.5% (P<0.01), respectively. The response of the HT-29 cells to celecoxib was similar to the MTT results (Fig. 1B); at 72 h, the level of GLI1 had decreased by 38.1% (P<0.05). However, the LoVo cells were resistant to the anticancer effect of celecoxib, which was also consistent with MTT results (Fig. 1B); following 72 h of treatment, GLI1 was decreased by only 9.6% (P>0.05). When the PANC-1 cells were treated with celecoxib, the GLI1 level also decreased by 15.5% (P<0.05) following 48 h of treatment.

### Effect of cyclopamine or celecoxib on the expression of PTCH1, SMO and GLI1 genes in colon cancer cells

**Figure 3:**
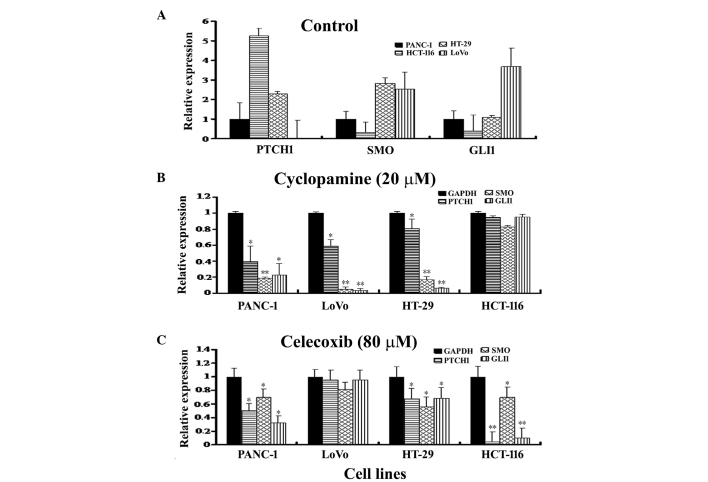
Effects of cyclopamine or celecoxib on the expression of *PTCH1*, *SMO* and *GLI1* genes in colon cancer cells by quantitative polymerase chain reaction analysis. (A) The graph presents the relative levels of *PTCH1*, *SMO* and *GLI1* mRNA in colon cancer cells, normalized against PANC-1 cells. The expression of *PTCH1*, *SMO* and *GLI1* genes was determined in PANC-1 and colon cancer cells treated with (B) cyclopamine or (C) celecoxib for 36 h. The relative levels of these genes were normalized against the control. ^*^P<0.05 and ^**^P<0.01 vs. control group. *GLI1*, glioma-associated oncogene homolog-1.

Based on the aforementioned results, the expression of the *PTCH1*, *SMO* and *GLI1* genes in the four cell lines was measured using qPCR (Fig. 3A). *PTCH1* was highly expressed in the HCT-116 cells, moderately expressed in the HT-29 and PANC-1 cells and poorly expressed in the LoVo cells. The mRNA levels were recorded as 5.26 (HCT-116), 2.29 (HT-29) and 0.03 (LoVo) comparerd with the internal control, *GAPDH*, when normalized against the PANC-1 cells. The *SMO* gene was highly expressed in the HT-29 and LoVo cells, however, a low expression level was observed in the HCT-116 cells, with mRNA levels of 2.81, 2.55 and 0.32, respectively, when normalized against the PANC-1 cells. *GLI1* expression was observed to be relatively low in the HCT-116 cells, moderate in the HT-29 and PANC-1 cells and high in the LoVo cells. The mRNA level for *GLI1* was 0.38 (HCT-116), 1.09 (HT-29) and 3.68 (LoVo) compared with the internal control, *GAPDH*, when normalized against the PANC-1 cells.

Following cyclopamine treatment, the LoVo cells were the most sensitive to cyclopamine treatment, as shown in Fig. 3B. The expression of *PTCH1*, *SMO* and *GLI1* mRNA was reduced to 58.9, 4.59 and 3.25% in the LoVo cells, respectively, compared with the control. These findings were consistent with the results shown in Figs. 1A and 2A. Cyclopamine effectively reduced the expression of *PTCH1*, *SMO* and *GLI1* mRNA to 39.7, 18.8 and 22.5% in the PANC-1 cells, and to 80.7, 16.5 and 6.37% in the HT-29 cells, respectively, compared with the control. However, the effect of cyclopamine on the expression of the genes in the HCT-116 cells was weak; the expression of *PTCH1*, *SMO* and *GLI1* mRNA was reduced to 94.5, 82.7 and 95.3% (P>0.05), respectively, compared with the control. This was consistent with the results shown in Figs. 1A and 2A.

Conversely, the HCT-116 cells were observed to be extremely sensitive to celecoxib treatment; the expression of *PTCH1*, *SMO* and *GLI1* mRNA was reduced to 4.0, 69.8 and 9.4% in the HCT-116 cells (Fig. 3C), respectively, compared with the control, consistent with the results presented in Figs. 1B and 2B. Celecoxib reduced *PTCH1*, *SMO* and *GLI1* mRNA expression to 67.3, 55.8 and 68.5% in the HT-29 cells and to 50.2, 69.9 and 32.2% in the PANC-1 cells, respectively, compared with the control. However, the changes in gene expression were minor in the LoVo cells, with the expression of *PTCH1*, *SMO* and *GLI1* mRNA reduced to 95.1, 81.1 (P>0.05) and 95.1%, respectively, compared with the control, consistent with the results shown in Figs. 1B and 2B.

## Discussion

Although aberrant Hh signaling is indicated to be involved in endodermally-derived human cancers that account for 25% of human cancer-related mortalities (19), the role of Hh signaling in human colorectal cancers is not fully understood (6,7), and several studies have indicated that Hh signaling is inactive in colorectal cancer (8–10). The results of the present study showed that Hh signaling activity varies between colon cancer HT-29, LoVo and HCT-116 cells. When the colon cancer cells were treated with cyclopamine, the LoVo cells were the most sensitive to the drug among the three cell lines, and compared with the control PANC-1 cells. Examination of the cells under the microscope and analysis of the MTT assay confirmed these results, indicating that Hh signaling was highly active in the LoVo cells. To the best of our knowledge, this is the first study to report Hh signaling activity in LoVo cells. Aberrant Hh signaling in the LoVo cells was evidently associated with the absent expression of *PTCH1* in these cells, which is consistent with the results of a previous study (20). The results of the current study demonstrated that the absent expression of *PTCH1* in LoVo cells is associated with epigenetic changes, as the expression of *PTCH1* was present in these cells following treatment with cyclopamine or celecoxib. The HT-29 cells showed a certain level of response to cyclopamine treatment according to microscopic examination and MTT assay. The ELISA results also indicated that cyclopamine downregulated the expression of *GLI1* in the HT-29 cells, suggesting that Hh signaling is active in HT-29 cells. In addition, the results indicated that the Hh signaling activity in the HT-29 cells was similar to that in the PANC-1 cells, but lower than that in the LoVo cells. Several studies have also shown that HT-29 cells possess Hh signaling activity and respond to cyclopamine (7,21,22), however, contrasting results have been presented (9). In the present study, the HCT-116 cells lacked sensitivity to cyclopamine, a result that was confirmed by GLI1 ELISA, suggesting that Hh signaling activity is low in HCT-116 cells. The results revealed that the low Hh signaling levels observed in HCT-116 cells are associated with a high expression level of *PTCH1* and a low expression level of *SMO*, which was methylated (23). Several studies have shown that HCT-116 cells exhibit low Hh signaling activity and lack a significant response to cyclopamine (8,9). The present study results indicated that cyclopamine may be used as an adjuvant treatment agent for Hh signaling-positive colon cancer.

The differing Hh signaling activities reflect the various malignant potentials in these colon cancer cell lines. Among the cell lines under investigation, LoVo cells possess high metastatic potential, whereas HT-29 cells exhibit low metastatic potential, and HCT-116 cells have low invasive capacity (24). You *et al* (20) identified *PTCH1* expression in HT-29 cells, while the expression of *PTCH1* was absent in LoVo cells, indicating that the expression of *PTCH1* is inversely correlated with the metastatic potential of colon cancer cell lines. The results presented in the present study support the view that Hh signaling is closely correlated with the malignant behaviors of colorectal cancer.

In the present study, in order to study the effects of celecoxib on Hh signaling in colon cancer cells, the cancer cell lines were treated with celecoxib. The results demonstrated that colon cancer and PANC-1 cells exhibit different sensitivities to celecoxib. When the PANC-1 cells were treated with celecoxib, cell growth was inhibited and the levels of GLI1 were significantly decreased. When the three colon cancer cell lines were treated with celecoxib, the HCT-116 cells were the most sensitive. This result was further confirmed by GLI1 assay, suggesting that celecoxib may target HCT-116 cells via the SMO-independent modulation of GLI1 activity, as the HCT-116 cells were not sensitive to the SMO inhibitor, cyclopamine. A previous study also showed that celecoxib may widely regulate the expression of proteins in HCT-116 cells based on proteomic profiles, and degrade GLI1 by downregulating molecular chaperone activities, activating tumor suppressors and regulating the expression of peroxiredoxin I and creatine kinase, among others (25). In another study, a similar result showed that celecoxib induces the proteasome-dependent degradation of T-cell factor-1 and −4 in HCT-116 cells (26). In the present study, the LoVo cells were resistant to the anticancer effect of celecoxib, and the change in GLI1 levels was mild following celecoxib treatment, suggesting that Hh signaling is essential for maintaining the malignant behavior of LoVo cells. The HT-29 cells showed a certain level of response to celecoxib treatment, according to microscopic examination and MTT assay. The GLI1 assay also revealed that celecoxib downregulated the expression of *GLI1* in the HT-29 cells, suggesting that the anticancer effects of celecoxib on HT-29 cells may be due to interference with Hh signaling.

Wnt/β-catenin signaling is important in the carcinogenesis of colorectal cancers (2). The canonical Wnt/β-catenin signaling pathway is composed of Wnt, the Wnt receptor, frizzled, and the signaling molecule, β-catenin. In the absence of Wnt, β-catenin forms a destruction complex containing the tumor suppressor APC protein. This complex leads to the destruction of β-catenin and therefore, the Wnt target genes are not expressed. When Wnt binds to its receptor, frizzled, it leads to the disintegration of the destruction complex and the accumulation of β-catenin in the cytoplasm. Subsequently, β-catenin translocates to the nucleus, where it interacts with Tcf/Lef transcription factors to promote the transcription of Wnt target genes. Therefore, APC and β-catenin are the basic components involved in Wnt signaling. The association between Wnt/β-catenin and Hh signaling is complex, as well as cooperative and competitive (27).

The different responses to cyclopamine or celecoxib may reflect a variety of genetic backgrounds in the colon cancer cell lines. In HCT-116 cells, *APC* is the wild-type and the *CTNNB1* gene, which encodes β-catenin, is the mutant-type, while in LoVo and HT-29 cells, *APC* is the mutant-type and *CTNNB1* is the wild-type (28). HCT-116 cells are often used as a representative of constitutive Wnt signaling. We believe that LoVo cells may be used as a representative of constitutive Hh signaling in colon cancer cells, as Hh signaling is highly active in LoVo cells. Furthermore, HCT-116 cells are COX-2 deficient, while HT-29 and LoVo cells exhibit COX-2 activities (29,30).

In conclusion, the results reported in the present study indicated that Hh signaling is activated in LoVo cells and, to a lesser degree, in HT-29 cells, but that it is inactive in HCT-116 cells. The highly activated Hh signaling in LoVo cells is associated with the absence of *PTCH1* expression in these cells. Celecoxib may inhibit the growth of HCT-116 cells via the SMO-independent modulation of GLI1 activity. However, LoVo cells are resistant to the growth inhibition of celecoxib, which has little effect on Hh signaling in this cell line. Celecoxib inhibits the growth of HT-29 cells by partly inhibiting the activity of Hh signaling. These results suggest that cyclopamine and celecoxib are potential treatment options for the targeted therapy of colon cancer.
